# Author Correction: Rad52 prevents excessive replication fork reversal and protects from nascent strand degradation

**DOI:** 10.1038/s41467-019-10072-9

**Published:** 2019-05-01

**Authors:** Eva Malacaria, Giusj Monia Pugliese, Masayoshi Honda, Veronica Marabitti, Francesca Antonella Aiello, Maria Spies, Annapaola Franchitto, Pietro Pichierri

**Affiliations:** 1 Mechanisms, Biomarkers and Models Unit, Department of Environment and Health, Istituto Superiore di Sanità, Viale Regina Elena 299, 00161 Rome, Italy; 2 Department of Biochemistry, Carver College of Medicine, University of Iowa, 51 Newton Road 4-403 Bowen Science Building, Iowa City, IA 52242 USA; 3 Istituto Nazionale Biostrutture e Biosistemi, Viale delle Medaglie d’Oro, 305, 00136 Rome, Italy

**Keywords:** Stalled forks, Genomic instability, DNA damage and repair, Stalled forks

Correction to: *Nature Communications* 10.1038/s41467-019-09196-9, published online 29 March 2019.

The original version of this Article contained an error in Fig. 2. The immunofluorescence images in panel d were inadvertently replaced with duplicates of those in panel c during final assembly of the figure. This has been corrected in the PDF and HTML versions of the Article.

