# Supplementary material for: Rad52 prevents excessive replication fork reversal and protects from nascent strand degradation
Source: Nat Commun. 2019 Mar 29;10:1412. doi: 10.1038/s41467-019-09196-9 (PMC6441034; doi:10.1038/s41467-019-09196-9)
Supplement: Supplementary file 1 — Supplementary Information [file 41467_2019_9196_MOESM1_ESM.pdf]

Supplementary Information

RAD52 PREVENTS EXCESSIVE REPLICATION FORK REVERSAL AND PROTECTS FROM NASCENT STRAND DEGRADATION

Malacaria E. et al.

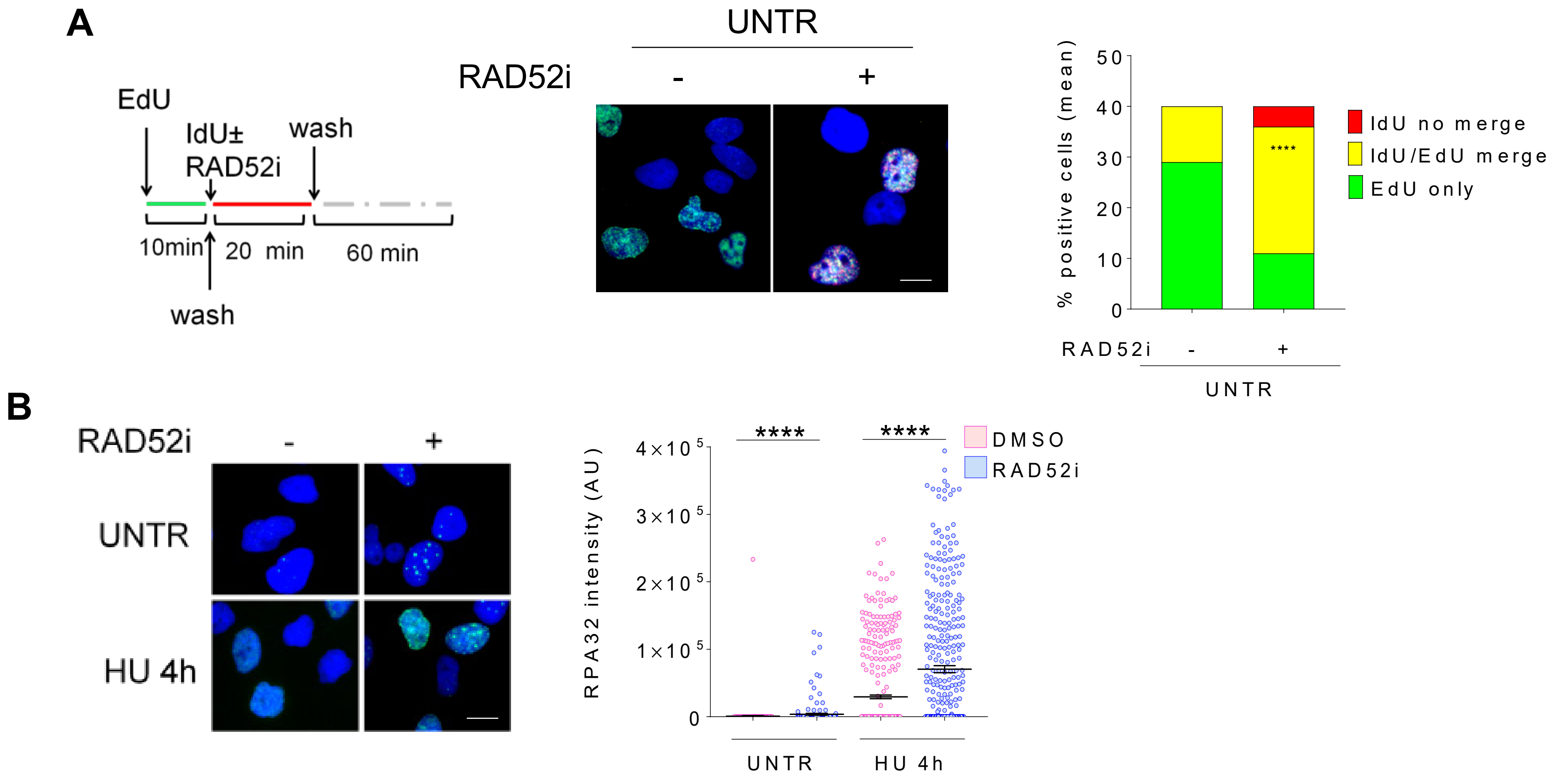

Supplementary Figure 1. Inhibition of RAD52 stimulates degradation of nascent strand by MRE11
A) Cells were labeled with IdU and EdU, as described in the scheme. EdU and IdU were used to detect nascent ssDNA and ongoing replication sites, respectively, in untreated cells. EdU was conjugated with Alexa Fluor 488 trough click-it reaction. IdU was detected under not-denaturating condition. Graph shows the percentage of cells positive for ssDNA (red), EdU (green) and both (yellow).N=200 Representative images are shown. **B**) Cells were treated as indicated, triton-extracted and subjected to anti-RPA32 immunostaining. Graph shows the main intensity of RPA32 staining for single nuclei from untreated or treated cells. RPA intensity was measured in at least 100 nuclei from two independent experiments. Values are presented as means ± SE (**** *P* < 0.0001; Mann–Whitney test N=307). Representative images of RPA32 immunofluorescence are shown. Scale bar is 5μm.

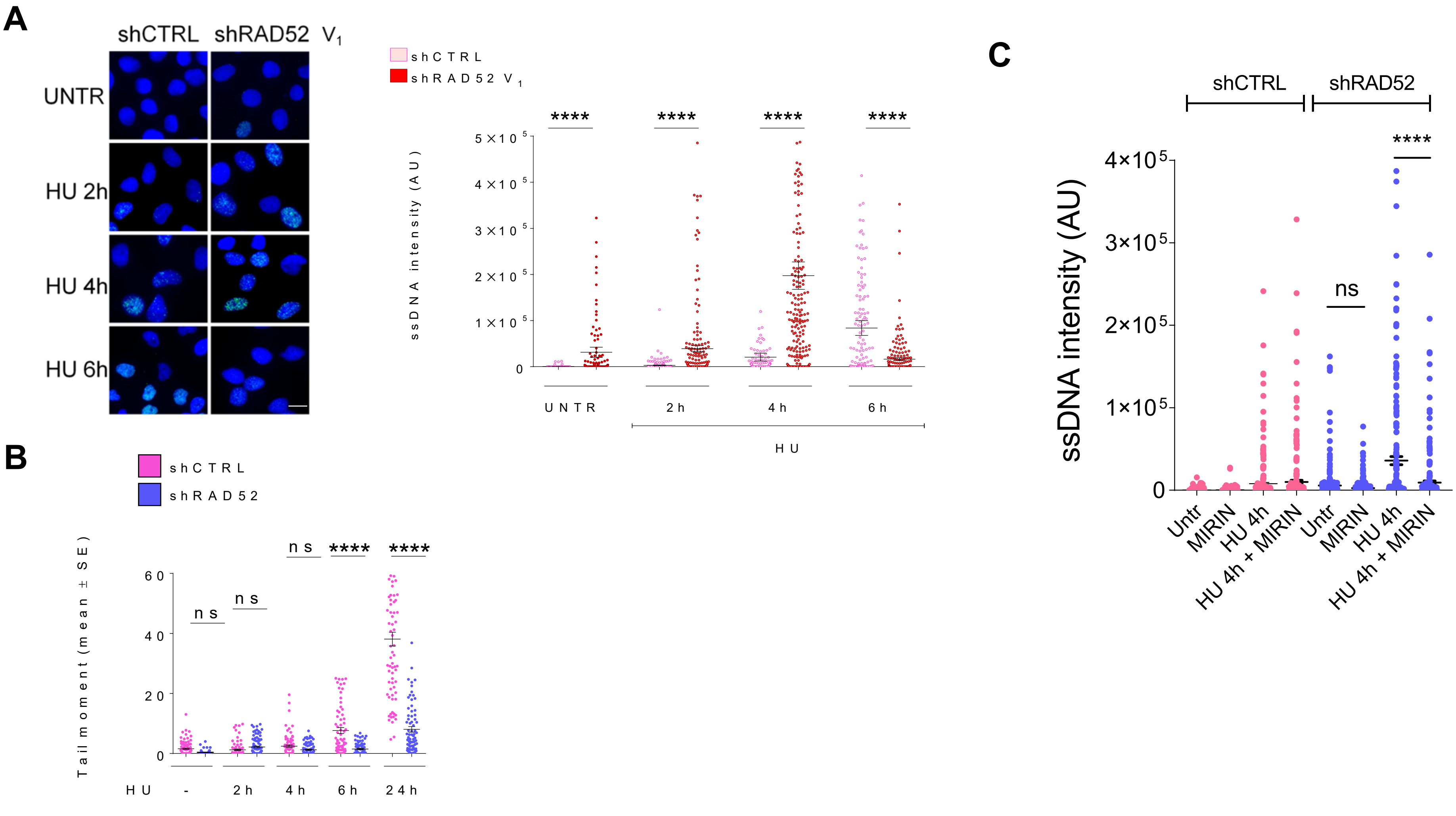

Supplementary Figure 2. Depletion of RAD52 by different shRNA recapitulates phenotypes of RAD52-inhibited cells

A) MRC5 WT (shCTRL) and MRC5 expressing the shRAD52 V_1 cassette (see Fig. 1A) were pulse labelled with IdU and treated with 2mM HU as indicated. Graph shows the main intensity of ssDNA staining for single nuclei. Values are presented as means ± SE (ns = not significant; **** $P <$ 0.0001; Mann–Whitney test N=195). Representative images are shown alongside. **B)** MRC5 WT (shCTRL) and MRC5 shRAD52 (V_2 cassette - see Fig. 1A) were pulse with 2mM HU for increasing time as indicated. The presence of DSBs was evaluated by neutral Comet assay. Graph shows the main tail moment. Values are presented as means ± SE (ns = not significant; **** $P <$ 0.0001; Student's t-test N=75).**C)** MRC5 WT (shCTRL) and MRC5 shRAD52 (V_2 cassette - see Fig. 1A) were pulse labelled with IdU and treated with HU for 4h in the presence or not of Mirin. Graph shows the main intensity of ssDNA staining for single nuclei. Values are presented as means ± SE (ns = not significant; **** $P <$ 0.0001; Mann–Whitney test N=280). Scale bar is 5μm.

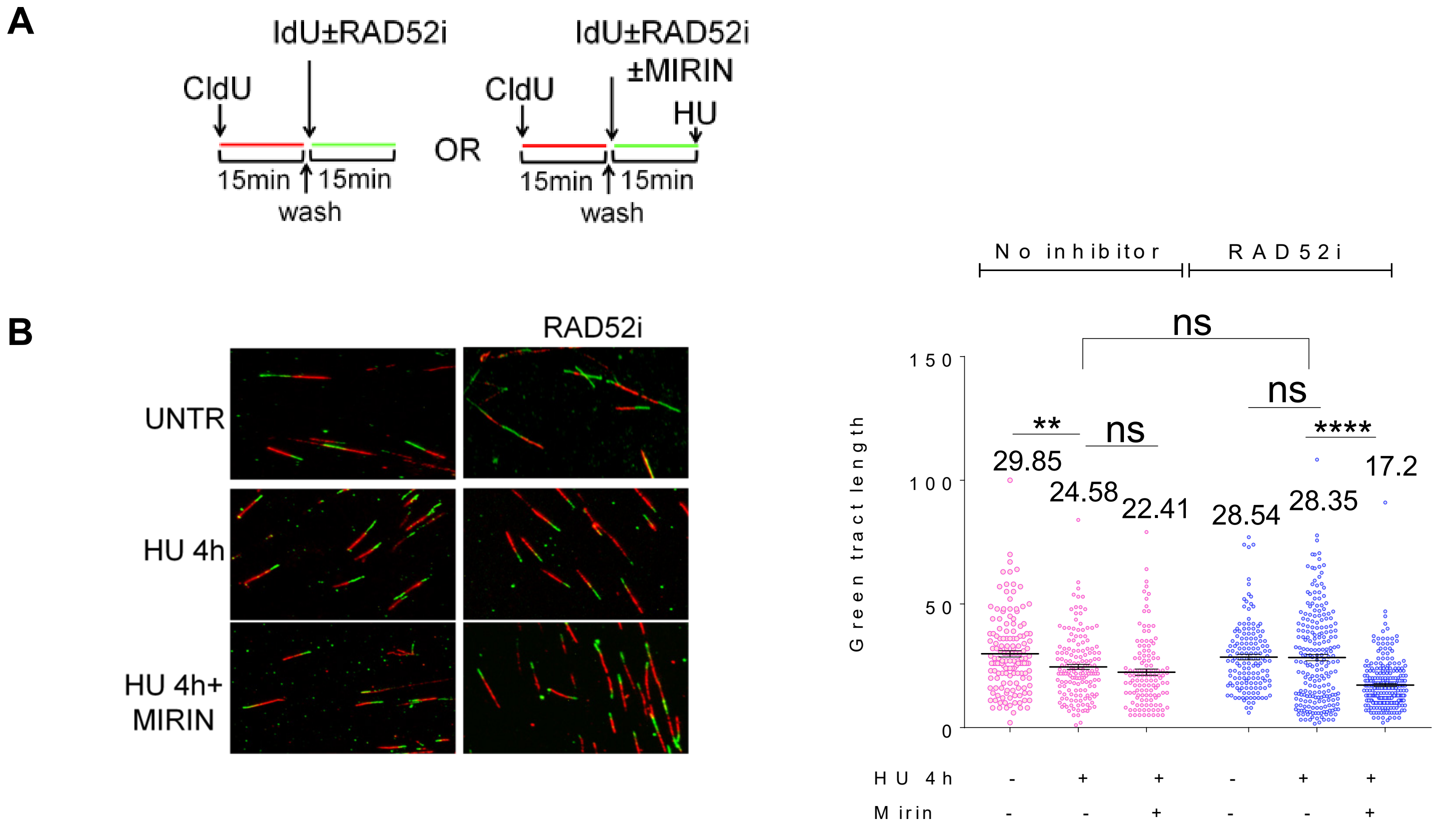

Supplementary Figure 3. Inhibition of RAD52 does not affect length of replication tracks
A) Schematic of DNA fibres experiment. **B**) Analysis of IdU tract length of ongoing forks. Dot plots show distribution of IdU tract lengths from single DNA fibres in MRC5 cells, after treatment with 2mM HU and in presence or not of RAD52i. When indicated, Mirin was added together with IdU, as in the experimental scheme. The length of the green tracks was measured in at least 100 well isolated DNA fibres from two independent experiments. Mean values are represented as horizontal black lines±SE and are reported on top of the scatter plot. Values are given in pixels. N=127 .Representative images of DNA fibres are shown. (ns = not significant; **** $P < 0.0001$; ANOVA test N=127).

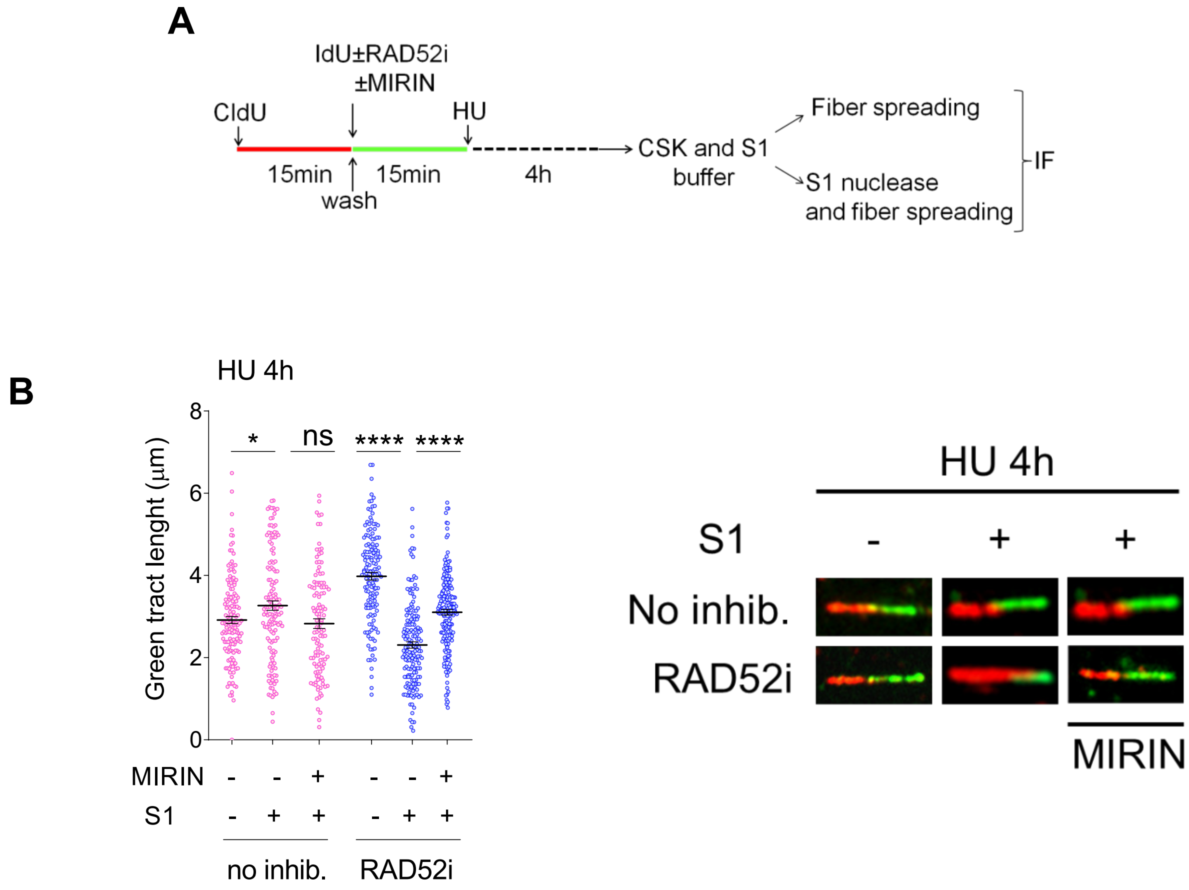

Supplementary Figure 4. Nascent DNA of replication forks from RAD52i cells contains ssDNA
A) Scheme of DNA fibers experiment treated or not with S1 nuclease. **B**) Analysis of IdU tract length of ongoing forks. Length of the green tracks were measured. Mean values are represented as horizontal black lines. Values are presented as means ± SE (ns = not significant; * *P* < 0.1**** *P* < 0.0001; Mann–Whitney test N=121). Representative images of single DNA fibers are shown.

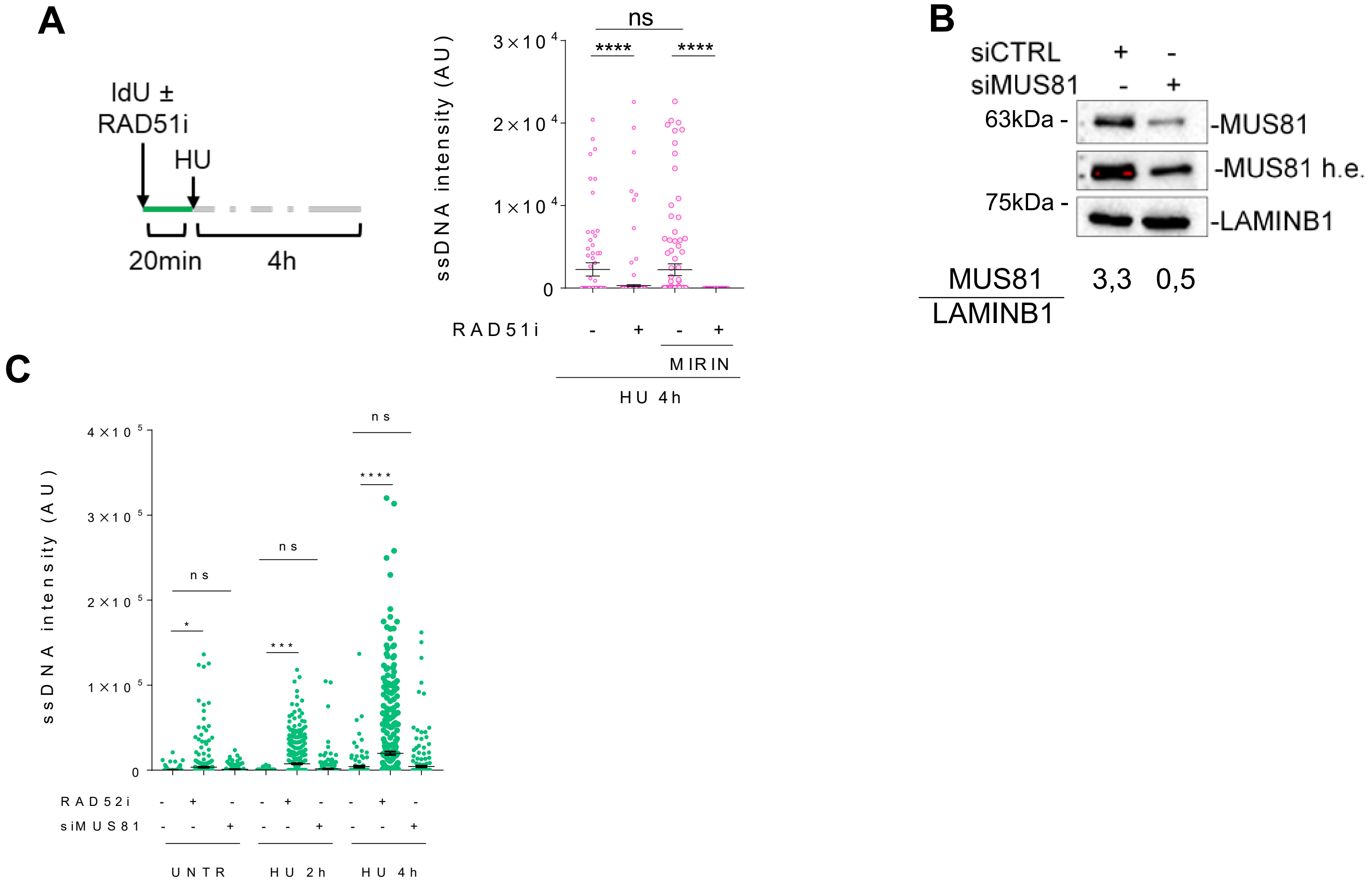

Supplementary Figure 5. The phenotype of RAD52i cells not recapitulates that conferred by RAD51 inhibition or MUS81 depletion
A) Scheme of the labelling protocol. Graph shows the mean intensity of ssDNA staining in at least 100 nuclei from two independent experiments. Values are presented as means ± SE (ns = not significant; * *P* < 0.1**** *P* < 0.0001; Mann–Whitney test N=273). **B**) Cells were transfected with scrambled siRNA (siCTRL) or siRNA directed against MUS81. Cell lysates were subjected to immunoblotting with indicated antibodies. Lamin B1 (LAMIN) was used as loading control. **C**) Analysis of nascent ssDNA in cells transfected with siCTRL and siMUS81. Data shows the main intensity of ssDNA staining for single nuclei from untreated or treated cells. At least 100 nuclei were analyzed from two independent experiments. Values are presented as means ± SE (ns = not significant; * *P* < 0.1 ;**** *P* < 0.0001; Mann–Whitney test N=549).

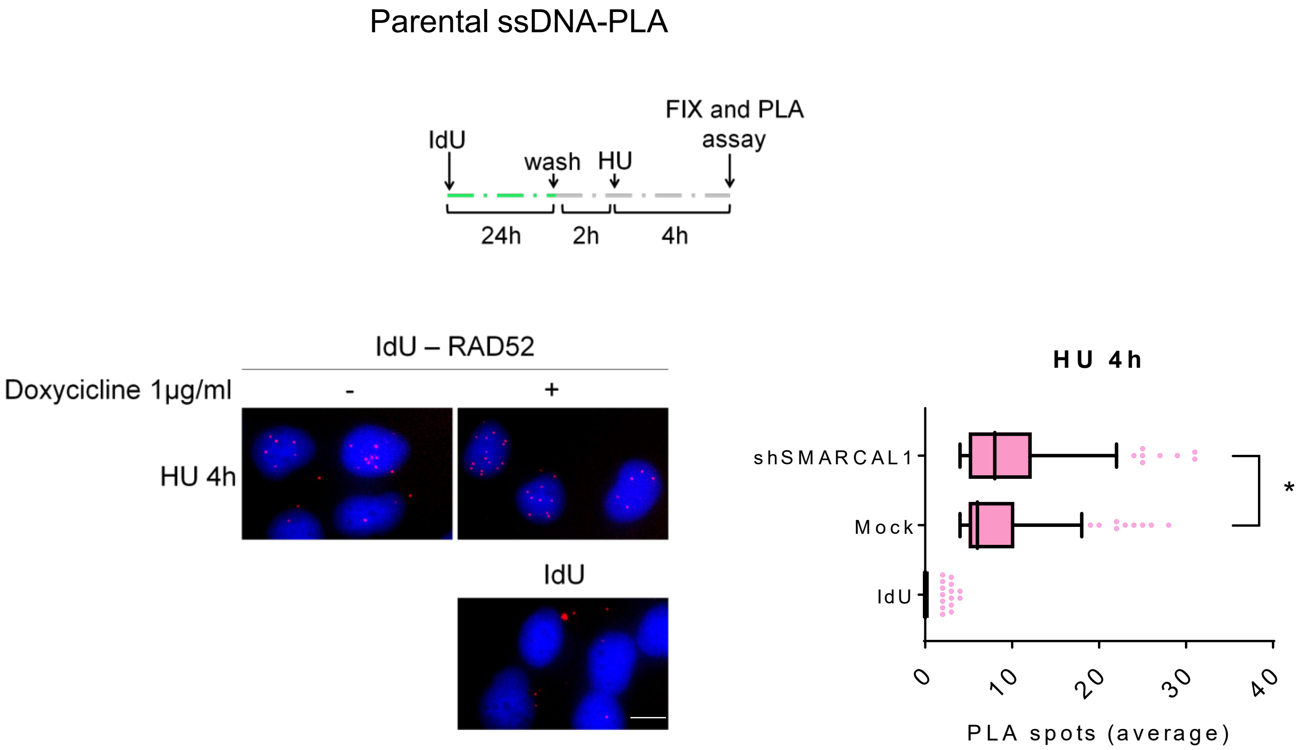

Supplementary Figure 6. Interaction of RAD52 with parental ssDNA is not affected by depletion of SMARCAL1

Cells depleted (shSMARCAL1; + doxycycline) or not (Mock; - doxycycline) of SMARCAL1 (see Fig. S7A) were cultured for 24h in the presence of IdU to label parental DNA and treated with HU as in the scheme on top. After 4h, cells were subjected to ssDNA-PLA with anti-IdU and anti-RAD52 antibodies. Graph shows the number of PLA spots in at least 100 nuclei from two independent experiments. Values are presented as means ± SE (* $P < 0.1$; Mann–Whitney test N=134). Representative images are shown. As a negative control for the PLA, cells were incubated with only the anti-IdU antibody (IdU). Scale bar is 5μm.

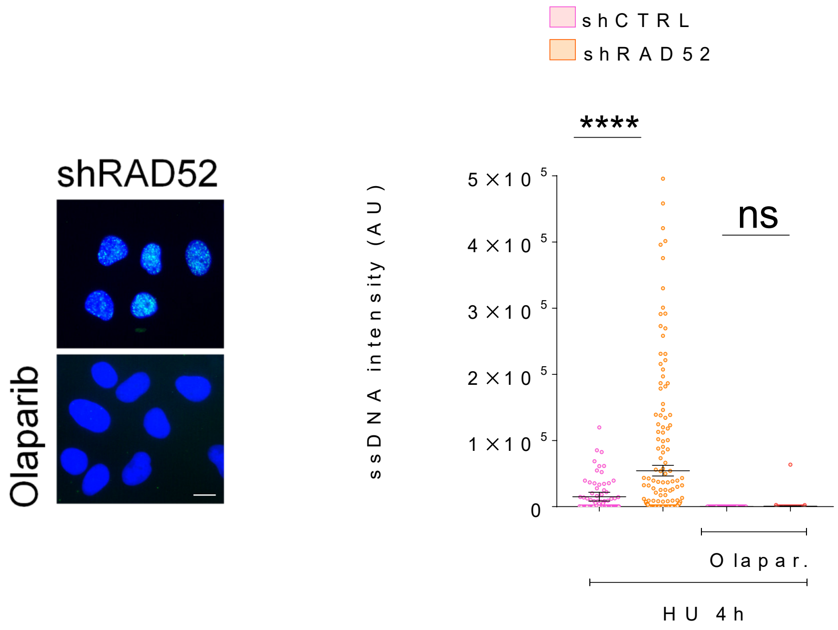

Supplementary Figure 7. Inhibition of PARP before treatment with HU suppresses formation of nascent ssDNA in shRAD52 cells
MRC5 stably-transduced with the shCTRL or shRAD52 (V2) virus were pulse-labelled with IdU prior to being treated with HU. Olaparib was added immediately before HU. Graph shows the mean intensity of ssDNA staining in at least 100 nuclei from two independent experiments. Values are presented as means ± SE (ns = not significant; **** $P < 0.0001$; Mann–Whitney test N=200). Representative images from shRAD52 cells are shown. Scale bar is 5μm.

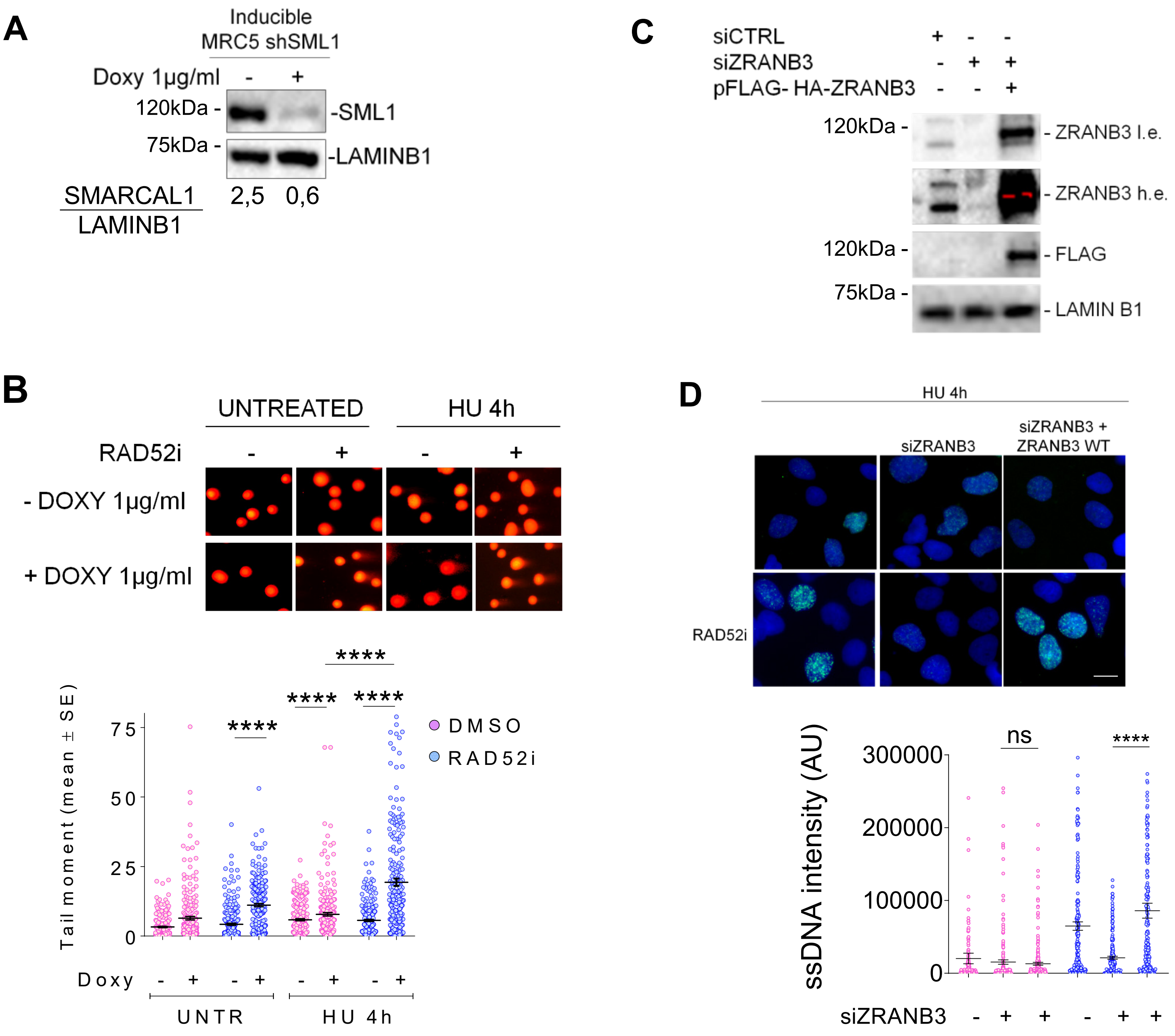

see next page

E

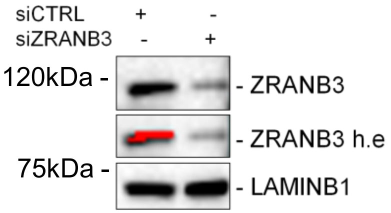

F

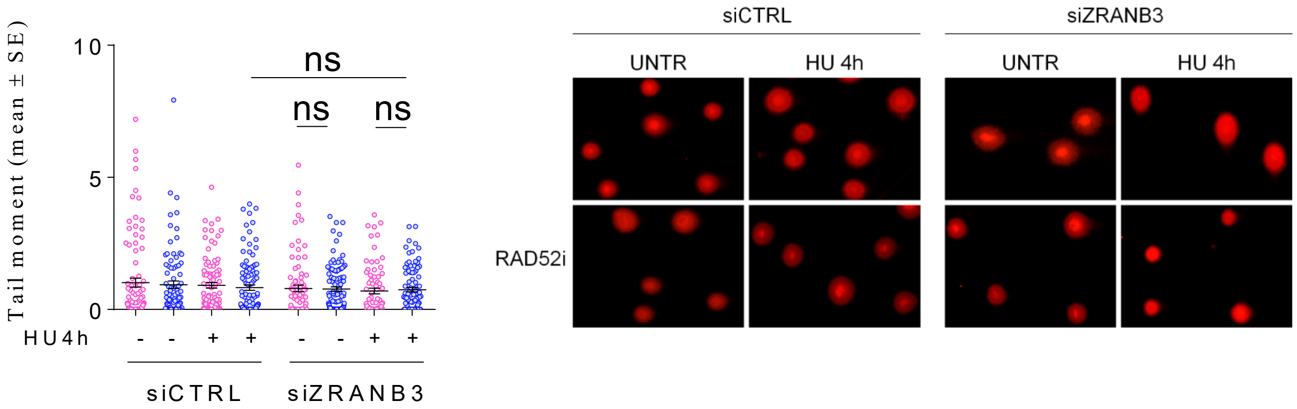

Supplementary Figure 8. Effect of SMARCAL1 or ZRANB3 depletion in RAD52i cells

A) Cells were infected with tetracycline-inducible virus containing shRNA sequences direct against SMARCAL1 to produce MRC5 shSMARCAL1 inducible cells lines. Cells were treated with doxycycline for 40 hrs then was collected and used to perform immunoblotting analysis and neutral comet assay. Immunoblotting shows level of SMARCAL1 protein. Lamin B1 was used as loading. **B**) Analysis of DSBs formation by neutral comet assay. Graph shows the mean tail moment±SE from two independent experiments. Representative images are shown. (**** p < 0.0001; Student's t-test N=239). **C**) Cells were transfected or not with pFLAG-HA-ZRANB3. After 6hrs cells were splitted and transfected with scrambled siRNA (siCTRL) or siRNA directed against ZRANB3.Cell lysates were subjected to Immunoblotting with indicated antibodies. LAMIN B1 was used as loading control. **D**) Dot plot shows the mean intensity of ssDNA staining for single nuclei. The intensity of the anti-IdU immunofluorescence was measured in at least 100 nuclei. Values are presented as means ± SE (ns = not significant; * *P* < 0.1 ;**** *P* < 0.0001; Mann–Whitney test N=197). Representative images are shown. **E**) Cells were transfected with scrambled siRNA (siCTRL) or siRNA directed against ZRANB3. Western blotting shows level of ZRANB3 protein. LAMIN was used as a loading control. **F**) After 48hrs of transfection cells were treated or not with 2mM HU at different point and were collected to perform Neutral comet assay. Data are presented as mean tail moment±SE (ns = not significant; Mann–Whitney test N=73). Scale bar is 5μm.

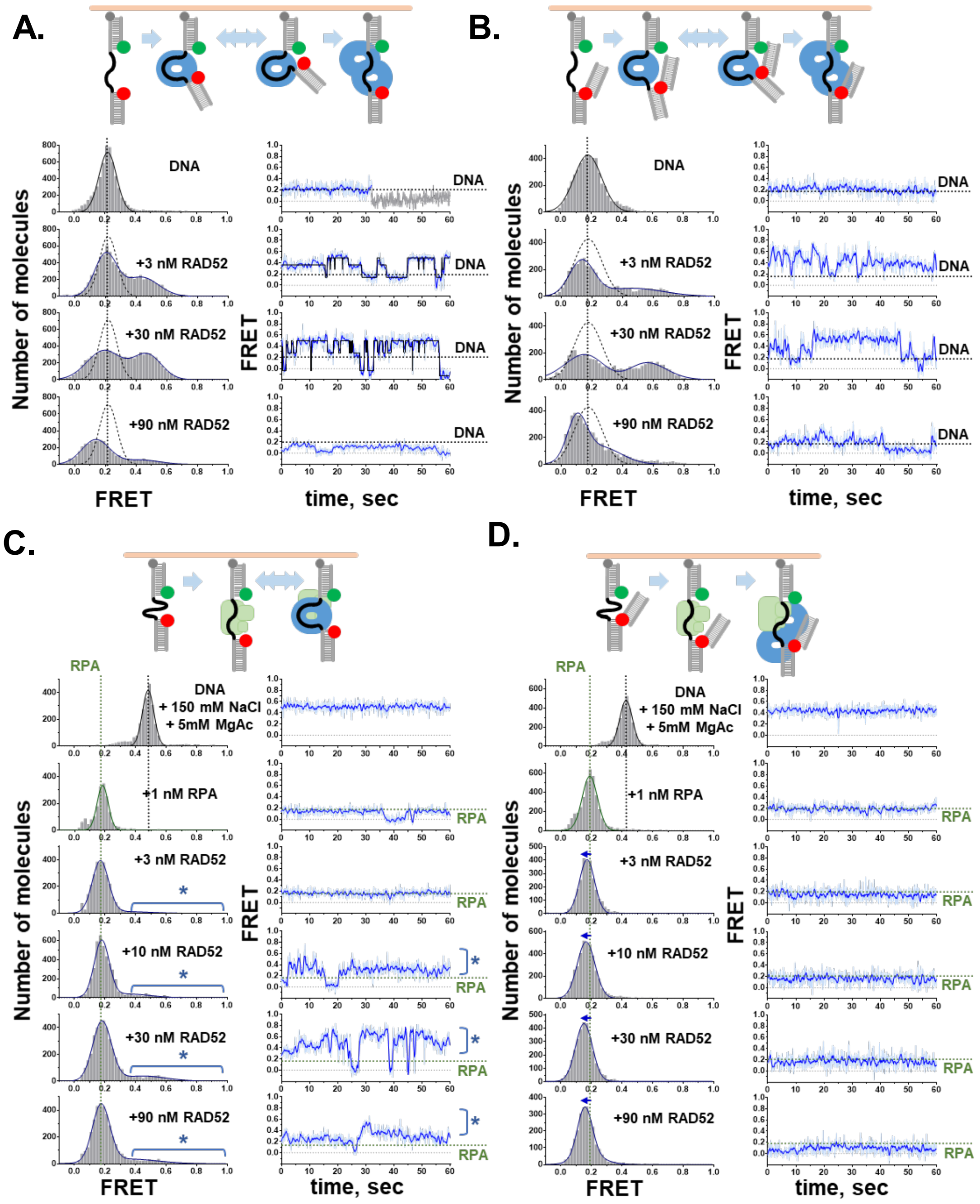

see next page

Supplementary Figure 9. RPA and the replication fork structure modulate the dynamics of the RAD52-ssDNA interaction
For all experimental conditions, DNA substrates were immobilized on the surface of the TIRFM flow cell and incubated with RPA and RAD52 as indicated. Several short movies were collected to assess the overall distributions of FRET states available to the ssDNA (black line) within each substrate. The resulting FRET distribution histograms are shown on the left. Dotted Gaussian picks correspond to the FRET distributions of the DNA (black) or DNA-RPA complex (green) in the absence of RAD52. Longer, 1 min movies were then collected to access conformational dynamics of the substrates. Representative trajectories are shown on the right of each distribution. **A-B**). Single-molecule FRET based analysis of the RAD52 dynamic interaction with a gapped (G1) and a fork-like DNA (RF1) substrates, respectively. For both substrates, we monitored the conformational rearrangement of the ssDNA gap in G1 (**A**) and RF1 (**B**), respectively. **A**). G1 DNA contains a 30 nt poly(dT) ssDNA gap flanked by two dsDNA arms 18 bp each. Each duplex is decorated with either Cy3 (green circle) or Cy5 (red circle) fluorescence dye. The substrate is bound to neutravidin on the pegylated flow cell surface through the biotin moiety (grey circle) on the 3' end of the long oligo. In low ionic strength, binding of the RAD52 results is wrapping of ssDNA around the RAD52 oligomeric ring, which is reflected in high FRET between Cy3 (FRET donor) and Cy5 (FRET acceptor) fluorophores (REF1 below). At higher RAD52 concentration the ssDNA is shared between multiple RAD52 rings, which results in the ssDNA extension and a low FRET signal. **B**). The RF1 substrate is similar to that in A, except for the presence of an additional DNA duplex representing a leading strand arm of the replication fork. Similar to the G1 substrate, we observed conformational dynamics associated with the wrapping of the ssDNA around the RAD52 ring. However, initial FRET for the RF1 substrate (0.18) was lower than that observed for G1 (0.2) suggesting that the Y structure of the fork favors slightly more extended conformation of the ssDNA region. RAD52 promoted wrapping of the ssDNA in the RF1 structure, but to a lesser degree than that on the gap; fewer trajectories displayed the dynamic behavior for this substrate. **C-D**). Single-molecule FRET based analysis of the RAD52 dynamic interaction with a gapped and a fork-like DNA (RF1) substrates in the presence of RPA, respectively. RPA binding to the 30 nt ssDNA gap on these substrates extends the ssDNA resulting in a low FRET state. **C**). At higher concentrations, RAD52 is able to wrap the RPA-ssDNA complex on the gap substrate. **D**). No dynamic wrapping/unwrapping interaction is observed for the RF1-RPA complex. The presence of both RPA and RAD52, however, shifted FRET distributions towards lower values suggesting that both RPA and RAD52 simultaneously interact with ssDNA causing in its further extension.

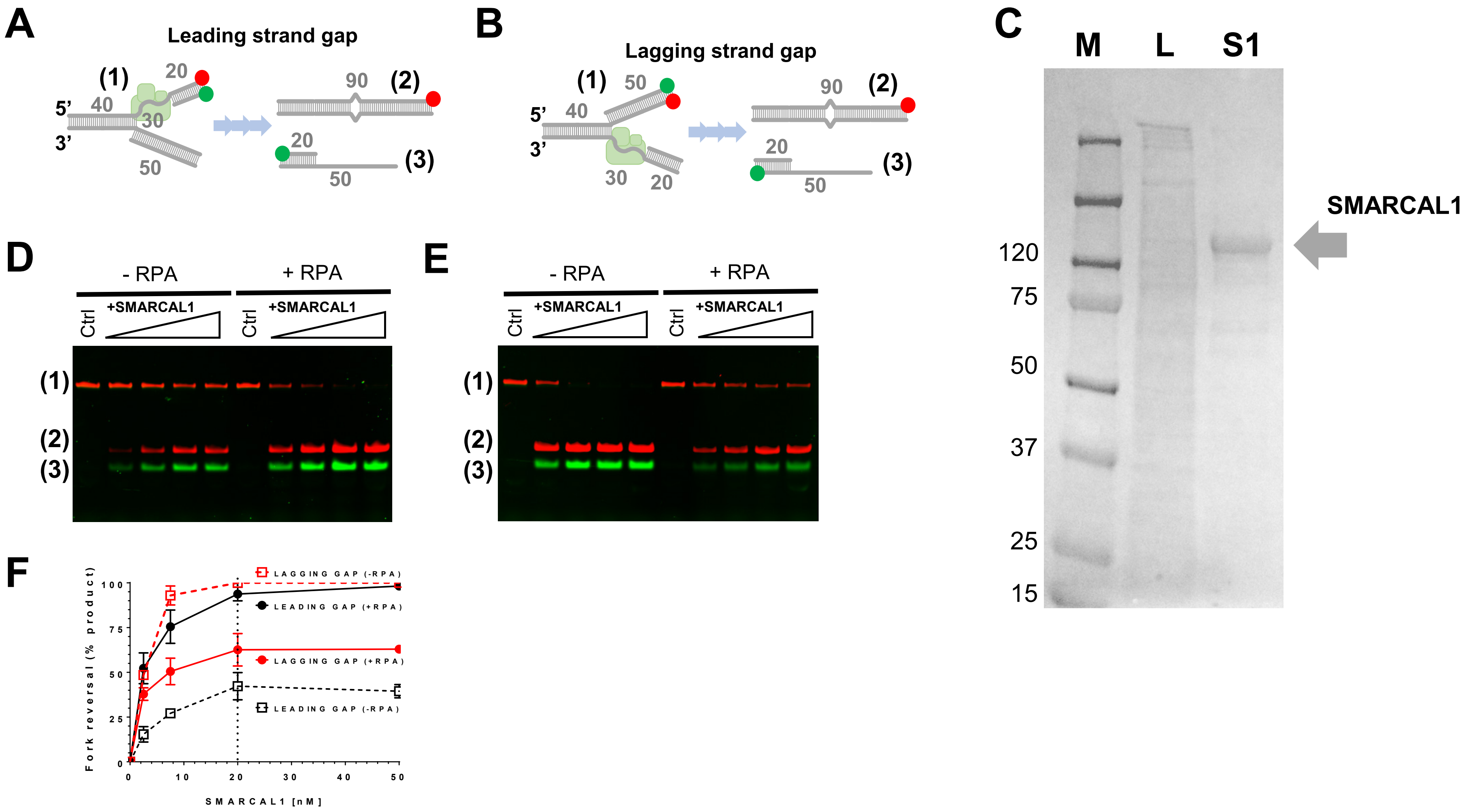

Supplementary Figure 10. Fork reversal by SMARCAL1. A – B. Cartoon depiction of the DNA substrates and the experimental scheme for the SMARCAL1-mediated fork reversal reaction. Synthetic fork structures used in these experiments contained a 30 nt ssDNA gap on the leading (**A**) and lagging (**B**) strand, respectively. Oligonucleotides used to construct these substrates are listed in the supplemental table S1. The lengths of the dsDNA and ssDNA features are marked in grey. Green circles depict Cy3 dyes, red circles depict Cy5 dyes. The substrate and the products of the fork reversal reactions are separated on the agarose gels after deproteinazation. **C)**. Gradient SDS-PAGE gel showing purified SMARCAL1. M –molecular weight markers, L – cell lysate, S1 – purified SMARCA1-Flag. **D).** Representative gel depicting SMARCA1-mediated fork reversal for the leading stand gap substrate in the presence and absence of RPA. Reactions were stopped at 15 min. **E**). The same as in (**C**), but for the fork with a lagging stand gap. **F**). The fraction of the reversed fork was quantified based on the disappearance of the substrate. Only Cy5 channel data were used for quantification. The data are presented as average and standard deviation for two independent experiments. Vertical line indicates SMARCA1 concentration at which the experiments in Fig. **5** were carried out.

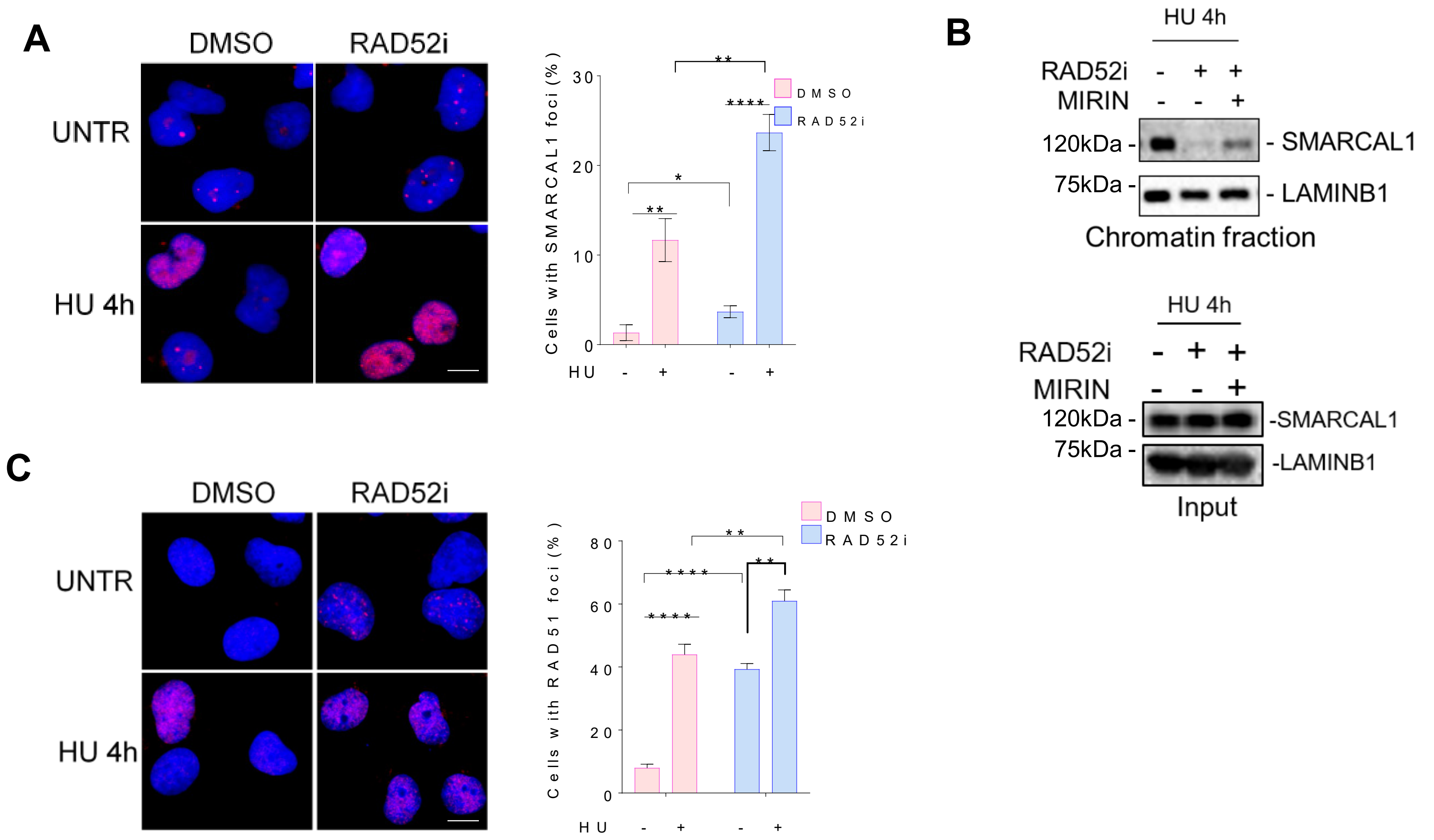

Supplementary Figure 11. Analysis of RAD51 and SMARCAL1 localization in nuclear foci

A) Cells were treated with 2mM HU, triton-extracted and subjected to anti-SMARCAL1 immunostaining. Graph shows the percentage of SMARCAL1 positive cells. Values are presented as means ± SE (*, p < 0.1; ** P < 0.01;**** P < 0.0001; Mann–Whitney test N=180). Representative images of SMARCAL1 immunofluorescence are shown. **B**) Chromatin recruitment of SMARCAL1 after replication stress in presence or not of RAD52 and or MIRIN. Lamin B1 was used as a loading control. **C**) Cells were treated as indicated, fixed and subjected to anti-RAD51 immunostaining. Graph shows the percentage of RAD51 positive cells. Values are presented as means ± SE (**, p < 0.01; *** P < 0.001;**** P < 0.0001; Mann–Whitney test N=230). Scale bar is 5µm.

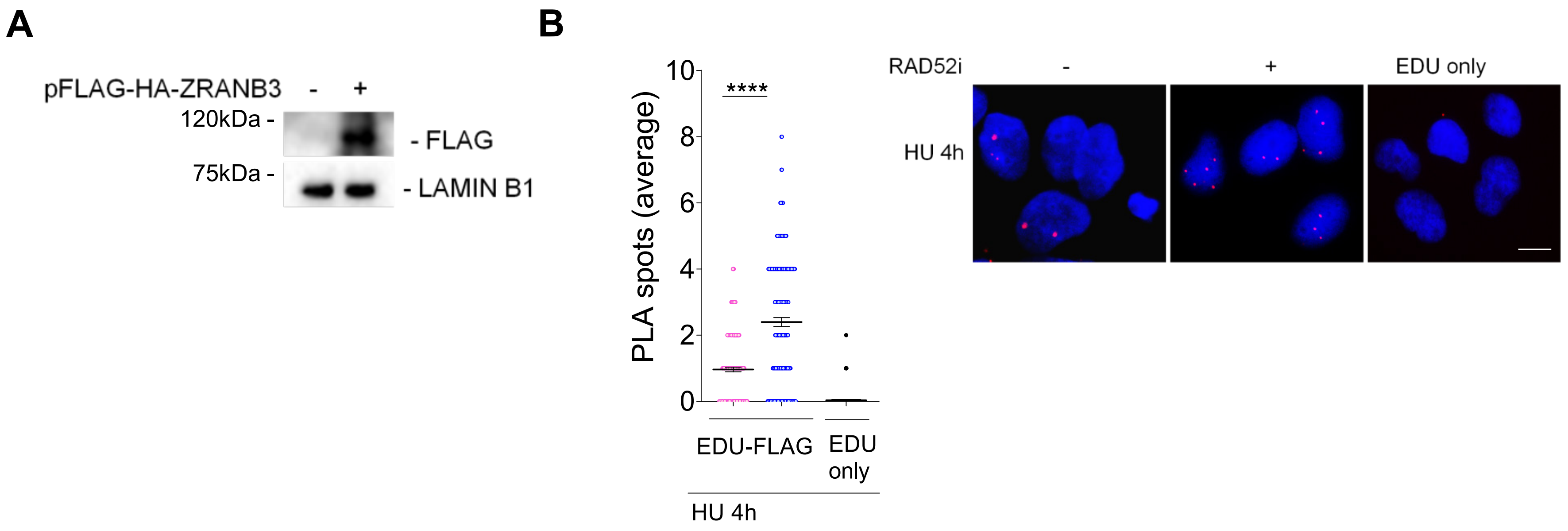

Supplementary Figure 12. Analysis of ZRANB3 fork recruitment

A) Western blot Analysis of pFLAG-ZRANB3 transfection. LAMIN was used as a loading control. **B**) Analysis of dsDNA–protein interaction by *in situ* PLA assay. DNA was labeled with EdU and Biotin was conjugated through Clik-it reaction. Antibodies raised against Biotin and FLAG were used to reveal EdU or ZRANB3, respectively. Immunofluorescence was performed in a non-denaturing condition. Cells stained with only anti-BIOTIN do not show PLA spots (negative control). Graph shows that PLA spots were not found. The graph shows the mean number of PLA spots per cell ± SE. Values are presented as means ± SE (**** $P < 0.0001$; Mann–Whitney test N=217). Scale bar is 5μm.

.

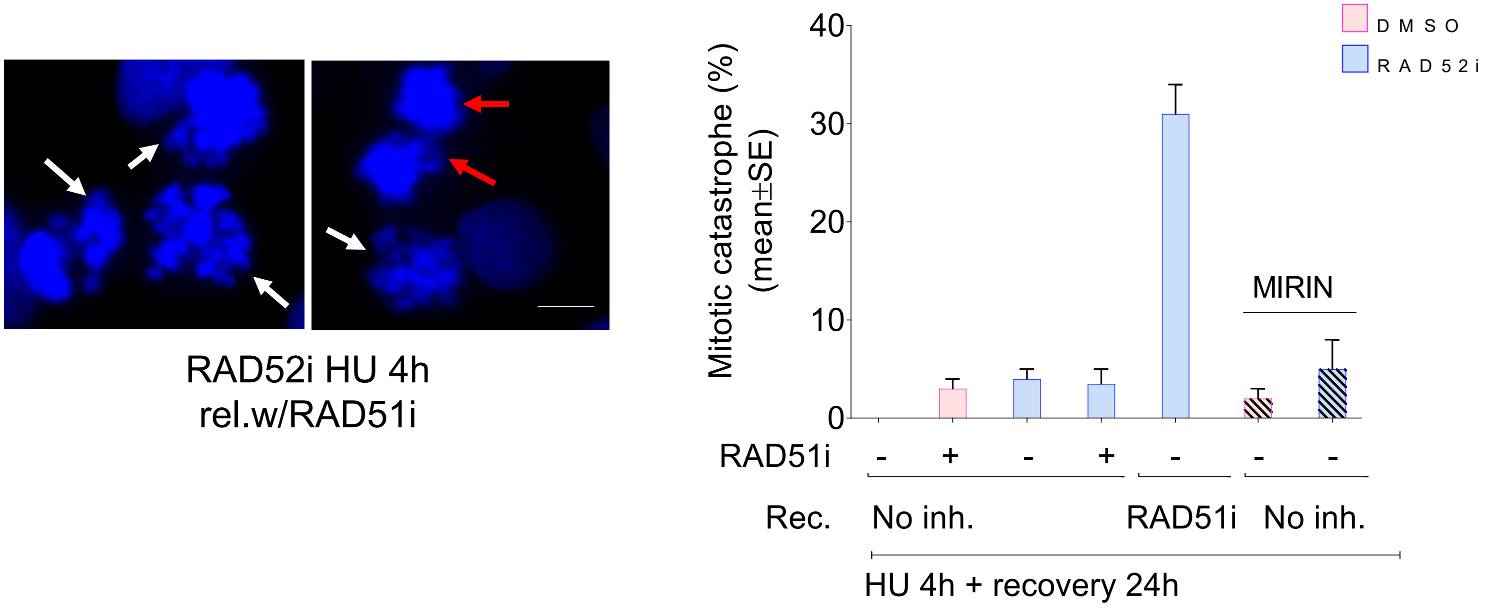

Supplementary Figure 13. Inhibition of RAD51 during recovery causes mitotic catastrophe in absence of a functional RAD52
Cells were treated as indicated and analysed for nuclear morphology by DAPI staining. An example of mitosis scored as mitotic catastrophe is shown in the images (white arrows denote examples of mitotic catastrophes while red arrows indicate normal mitosis). (ns, not significant; ** $P < 0.01$; **** $P < 0.0001$; Mann–Whitney test N=250). Scale bar is 5μm.

Supplementary Table 1. Oligonucleotides used in this study

Oligonucleotide	Sequence and modifications
**#1**	5'-(bio)-CTC AAG CCA TCC GCA ACG TTT TTT TTT TTT TTT TTT TTT TTT TTT TTT GAA ACA AAG GGC TCC TCA-3'
**#2**	5'-(Cy3)-CGT TGC GGA TGG CTT GAG-3'
**#3**	5'-TGA GGA GCC CTT TGT TTC-(Cy5)-3'
**#4**	5'-TGA GGA GCC CTT TGT TTC TGG CGA CGG CAG CGA GGC ATC TGA TAT GCA-3'
**#5**	5'-(Cy5)-TGC ATA TCA GAT GCC TCG CTG CCG TCG CCA-3'
**#6**	5'-TGC ATA TCA GAT GCC TCG CTG CCG TCG CCA-(Cy5)-3'
**#7**	5'-TGC ATA TCA GAT GCC TCG CTG CCG TCG CCA-3'
**#8**	5'-(Cy5)-TGA GGA GCC CTT TGT TTC TGG CGA CGG CAG CGA GGC ATC TGA TAT GCA-3'
**90TOP***	5'- CGT GAC TTG ATG TTA ACC CTA ACC CTA AGA TAT CGG GTA TTC AGA GTG TGA GGA TAC GCA TAG GCA GAT GCA ACG TGT CTA TCA CCT GAA -3'
**90BOTCy3**	5'-(Cy3)- TTC AGG TGA TAG ACA CGT TGC ATC TGC CTA TGC GTA TCC TCA CAC TCT GAT AAC CCG ATA TCT TAG GGT TAG GGT TAA CAT CAA GTC ACG -3'
**50BOT**	5'-TTC AGG TGA TAG ACA CGT TGC ATC TGC CTA TGC GTA TCC TCA CAC TCT GA -3'
**20TOPCy5**	5'- CAA CGT GTC TAT CAC CTG AA -(Cy5)-3'
**20BOT**	5'- TTC AGG TGA TAG ACA CGT TG -3'
**50TOPCy5**	5'- TCA GAG TGT GAG GAT ACG CAT AGG CAG ATG CAA CGT GTC TAT CAC CTG AA -(Cy5)-3'

* The underlined nucleotides create a 2 bp mismatch on the parental strands to prevent spontaneous branch migration

Supplementary Table 2. Antibodies used in this study

Antibody	Company and product ID	Host	Applications		
			IF	PLA	WB
MUS81	Santa Cruz Biotechnology sc-47692	mouse			1 :2000
Lamin B1	Abcam AB16048	rabbit			1 :10000
GAPDH	Millipore MAB374	mouse			1: 5000
SMARCAL1	Abcam AB154226	rabbit			1 :1000
RPA32	Millipore RPA34-20	mouse	1 :300		1 :1000
RAD51	Bioss	rabbit			1 :1000
ZRANB3	Proteintech	rabbit			1 :1000
RAD52	Aviva OAAF01156	rabbit		1 :150	
RAD51	Bioss BS-20297R	rabbit	1 :100	1: 100	1:1000
Biotin	Invitrogen 31852	mouse		1 :500	
BrdU	Becton Dickinson 347580	mouse		1: 10	
SMARCAL1	Abcam ab154226	rabbit	1 :100	1 :100	
MRE11	Bethyl A300-181A	rabbit		1 :1000	
BLM	Santa Cruz Biotechnology sc-7790	donkey	1 :50		
RPA32	Millipore	mouse	1: 100		
BrdU	Abcam AB16048	Rat	1: 60		
