# Supplementary material for: Rad52 prevents excessive replication fork reversal and protects from nascent strand degradation
Source: Nat Commun. 2019 Mar 29;10:1412. doi: 10.1038/s41467-019-09196-9 (PMC6441034; doi:10.1038/s41467-019-09196-9)
Supplement: Supplementary file 3 — Reporting Summary [file 41467_2019_9196_MOESM3_ESM.pdf]

nature research

Corresponding author(s): Pietro Pichierri

Last updated by author(s): Feb 11, 2019

Reporting Summary

Nature Research wishes to improve the reproducibility of the work that we publish. This form provides structure for consistency and transparency in reporting. For further information on Nature Research policies, see Authors & Referees and the Editorial Policy Checklist.

Statistics

For all statistical analyses, confirm that the following items are present in the figure legend, table legend, main text, or Methods section.

n/a	Confirmed	
☐	☒	The exact sample size (*n*) for each experimental group/condition, given as a discrete number and unit of measurement
☐	☒	A statement on whether measurements were taken from distinct samples or whether the same sample was measured repeatedly
☐	☒	The statistical test(s) used AND whether they are one- or two-sided<br>*Only common tests should be described solely by name; describe more complex techniques in the Methods section.*
☒	☐	A description of all covariates tested
☒	☐	A description of any assumptions or corrections, such as tests of normality and adjustment for multiple comparisons
☒	☐	A full description of the statistical parameters including central tendency (e.g. means) or other basic estimates (e.g. regression coefficient) AND variation (e.g. standard deviation) or associated estimates of uncertainty (e.g. confidence intervals)
☐	☒	For null hypothesis testing, the test statistic (e.g. *F*, *t*, *r*) with confidence intervals, effect sizes, degrees of freedom and *P* value noted<br>*Give P values as exact values whenever suitable.*
☒	☐	For Bayesian analysis, information on the choice of priors and Markov chain Monte Carlo settings
☒	☐	For hierarchical and complex designs, identification of the appropriate level for tests and full reporting of outcomes
☒	☐	Estimates of effect sizes (e.g. Cohen's *d*, Pearson's *r*), indicating how they were calculated

Our web collection on statistics for biologists contains articles on many of the points above.

Software and code

Policy information about availability of computer code

Data collection	Immunofluorescent data were acquired using the Image-Pro-Plus 6.0 software (Nikon). Blots and gels were acquired using the ImageLab software of the ChemiDoc MP apparatus (Bio-Rad) or through ImageJ. Single-molecule trajectories were extracted from the recorded video file by IDL software. Fluorescence trajectories were analyzed using customized MATLAB (The MathWorks, Inc.), scripts (available upon request from the Spies lab).
Data analysis	Prism 7 (GrphPad inc) or ORIGEN was used for graph generation and data analyses

For manuscripts utilizing custom algorithms or software that are central to the research but not yet described in published literature, software must be made available to editors/reviewers. We strongly encourage code deposition in a community repository (e.g. GitHub). See the Nature Research guidelines for submitting code & software for further information.

Data

Policy information about availability of data

All manuscripts must include a data availability statement. This statement should provide the following information, where applicable:

- Accession codes, unique identifiers, or web links for publicly available datasets
- A list of figures that have associated raw data
- A description of any restrictions on data availability

The authors declare that all relevant data supporting the findings of this study are available within the article and its supplementary information files or from the corresponding author upon reasonable request. Data source files are presented as supplemental information

Field-specific reporting

Please select the one below that is the best fit for your research. If you are not sure, read the appropriate sections before making your selection.

☒ Life sciences ☐ Behavioural & social sciences ☐ Ecological, evolutionary & environmental sciences

For a reference copy of the document with all sections, see nature.com/documents/nr-reporting-summary-flat.pdf

Life sciences study design

All studies must disclose on these points even when the disclosure is negative.

Sample size	Sample size was not predetermined and was indicated in the figure legends. Sample size was selected on the basis of relevant experience in our lab and previous studies by our and others
Data exclusions	No data exlcusion was performed
Replication	All experiments were repeated at least 2 times, unless stated differently in figure legends. The main observations of the work were reproduced in different human cell lines, in different experimental settings and by different technologies. Sample size and number of independent experiments are clearly stated in the figure legend or in methods
Randomization	No randomization method was used
Blinding	Investigators were not blinded, however, critical experiments were analysed by independent investigators

Reporting for specific materials, systems and methods

We require information from authors about some types of materials, experimental systems and methods used in many studies. Here, indicate whether each material, system or method listed is relevant to your study. If you are not sure if a list item applies to your research, read the appropriate section before selecting a response.

Materials & experimental systems

n/a	Involved in the study	
☐	☒	Antibodies
☐	☒	Eukaryotic cell lines
☒	☐	Palaeontology
☒	☐	Animals and other organisms
☒	☐	Human research participants
☒	☐	Clinical data

Methods

n/a	Involved in the study	
☒	☐	ChIP-seq
☒	☐	Flow cytometry
☒	☐	MRI-based neuroimaging

Antibodies

Antibodies used	See supplementary Table 2
Validation	Validations are based on the datasheets from the manufactures. In addition, for some antibodies (RAD52, SMARCAl1 and ZRANB3) validation was also internal, derived from comparison between CTRL-siRNA and gene-siRNA treated samples, as shown also in the figures.

Eukaryotic cell lines

Policy information about cell lines

Cell line source(s)	MRC5SV40 are a gift from Patricia Kannouche (CNRS, UMR8200 IGR Villejuif, France); shRAD52 cell lines were generated in this study
Authentication	All cell lines derives from MRC5SV40, which was authenticated originally, but were not authenticated after generation
Mycoplasma contamination	All cell lines are mycoplasma-free as tested by DAPI-stain
Commonly misidentified lines (See ICLAC register)	No commonly misidentified cell lines were used
